# Group Belonging and Social Identities in the Transition of Asylum-Seekers in Greece: Longitudinal Pathways to Adjustment

**DOI:** 10.3390/healthcare12010055

**Published:** 2023-12-26

**Authors:** Angelos Panagiotopoulos, Vassilis Pavlopoulos

**Affiliations:** Department of Psychology, School of Philosophy, National and Kapodistrian University of Athens, 157 72 Athens, Greece; vpavlop@psych.uoa.gr

**Keywords:** asylum-seekers, forced migration, social identity change, adjustment, life change, SIMIC

## Abstract

Millions of forced migrants settling in host countries often struggle to adjust to their new life. As their inclusion and adjustment within receiving societies has become a global social challenge, studying the factors that support their successful transition is an important topic of research inquiry. The present three-wave longitudinal study examined the role of group belonging and social identification in facilitating the transition of 60 sub-Saharan African asylum-seekers to Greece. Drawing upon the Social Identity Model of Identity Change (SIMIC), we investigated how multiple group memberships before migration, social identity continuity, and social identity gain related to their adjustment over 8 months. On the between-person level, multiple group belonging before migration indirectly contributed to better person-average levels of sociocultural adjustment, physical health functioning and satisfaction, psychological distress, and life satisfaction, by way of higher person-average levels of social identity continuity and/or social identity gain. However, multiple groups before migration also had a direct negative effect on the overall levels of psychological distress. On the within-person level, positive changes in social identity continuity and gain were related to positive changes in different adjustment-related outcomes over time. Our findings are consistent with SIMIC and highlight the importance of group belonging and associated social identities in forced migrants’ transition, in ways that may pave the way for the development of social identity interventions to promote their health, well-being, and successful integration. Future longitudinal and experimental evidence with larger and more diverse samples of forced migrants is needed to establish the generalizability and causality of the observed associations.

## 1. Introduction

In recent decades, the global number of forced migrants has been on the rise, reaching 109 million by mid-2023 [1]. This number is projected to further increase in the near future as the main driving factors—war, violence, oppression, and sociopolitical unrest—are unlikely to cease [2].

Exposure to pre- and peri-migratory psychological trauma and adversity [3,4], as well as post-migratory stressors in receiving societies, including socioeconomic disadvantage [5], lengthy asylum procedures [6], family separation [7], social isolation [8], and discrimination [9], adversely affect the health, well-being, and sociocultural integration of forced migrants. Specifically, as a consequence of the above, forced migration has consistently been associated with very high rates of post-traumatic stress disorder; depression and anxiety disorders [3,4]; low levels of subjective well-being [10], life satisfaction [11], and self-esteem [12]; increased risk of physical limitations and chronic diseases [13,14]; and low levels of sociocultural adjustment [15].

In contrast to the study of the negative effects of forced migration and the various risk factors along the migration trajectory, much less research has focused on the ways that forced migrants may successfully transition and adjust within receiving societies. Although researchers advocate for greater social equality and the mitigation of post-migratory stressors through policy interventions ([16], e.g., employment and family reunification programs for refugees), on the psychological level, it is less clear which factors may protect forced migrants, and consequently what can be done to achieve better outcomes. As their long-term integration becomes a pressing social issue for host societies, this remains a research area of concern not only for the well-being of forced migrants themselves but also for the social cohesion of the host societies [17]. In this regard, guided by the Social Identity Approach to Health and the Social Identity Model of Identity Change in particular (SIMIC; for a review, see [18]), the present study seeks to investigate whether and how group belonging—and the associated social identities—may support the adjustment of forced migrants.

### 1.1. Social Identity Approach to Health and SIMIC

The Social Identity Approach to Health argues that social groups affect health and well-being through their capacity to be integrated into people’s self-concept in the form of social identities [19]. This is because social identification with groups provides basic psychological resources that are fundamental to adaptive functioning and good health. These resources include a feeling of connection, belonging, meaning, and worth; a perception of agency and collective efficacy; and the provision and reception of social support [20,21]. As such, social groups—through the identities attached to them—are thought to serve as social cures.

Building upon the basic premises of the Social Identity Approach to Health, the basic tenet of SIMIC is that major life transitions typically modify one’s group memberships and thus introduce some form of social identity change and reconstruction [18]. In particular, some groups may be left behind while other group memberships may be gained. However, this change process is inherently stressful because it threatens one’s sense of self. Since social identities provide basic psychological resources, one’s ability to cope with this threat to adjust and navigate effectively through life change is compromised [18].

Central to SIMIC is the notion that multiple group memberships before a major life transition serve a protective function; the more groups someone belongs to, the more psychological resources they will have at their disposal to cope and adjust successfully (e.g., [22]). The adjustment-related benefits of multiple group belonging derive from two distinct social identity pathways. First, the social identity continuity pathway, through which belonging to multiple groups prior to life change makes it all the more probable that some of these group memberships will be preserved following the transition (e.g., [23]). In turn, preserved group memberships provide a sense of continued selfhood and access to key group-based psychological resources. Second, adjustment is facilitated by forming new group memberships and corresponding social identities through the social identity gain pathway (e.g., [24]). Belonging to multiple groups before a significant life transition contributes to this process by offering a lived experience of group life and sociopsychological capital upon which individuals can build new memberships and identities.

Evidence speaking to the key predictions of SIMIC comes from a variety of life transitions, including pursuing tertiary education [25], shifting into retirement [24], motherhood [26], recovering from addiction [27], recovering from depression [28], the onset of chronic diseases [23], and adjusting to psychological trauma [29].

### 1.2. SIMIC and Forced Migration

Forced migration represents a very significant life change. Settling as a forced migrant in a new country invariably threatens one’s group memberships and thus sense of self. This often entails an interruption of relations with family and friends, the loss of professional and social roles, and threats to one’s ethnic, cultural, and religious identities in the post-migratory context. Extrapolating from the key insights of SIMIC, belonging to multiple groups before migration would provide a platform for forced migrants to maintain existing pre-migratory group memberships and acquire new post-migratory ones in the receiving country. Accordingly, this process of identity reconstruction via the two social identity pathways would positively contribute to their adjustment.

Yet, the applicability of SIMIC to forced migration cannot be inferred with certainty. First, the vast majority of forced migrants originate from non-WEIRD (western, educated, industrialized, rich, and democratic) societies [30], whereas evidence for the main assumptions of the Social Identity Approach to Health has been based on WEIRD samples. In fact, the extent to which groups provide psychological resources may be moderated by cross-cultural normative differences [31]. Second, the experience of forced migration presents additional identity challenges compared to the more positive and/or voluntary transitions where SIMIC has mostly been applied (e.g., tertiary education, motherhood, addiction recovery). For instance, the refugee identity is often stigmatized and devalued within host societies, which in turn may undermine health and adaptive functioning [32]. In addition, identity continuity may not always be a viable route for forced migrants (e.g., due to the death of family members), while contextual barriers may preclude identity gain in the host society (e.g., inability to study or work due to legal status).

To our knowledge, only one study has tested quantitatively part of SIMIC among forced migrants. In their cross-sectional study, Smeekes et al. [33] focused on multiple group memberships and the continuity pathway among Syrian refugees in Turkey. Refugees belonging to multiple social groups before fleeing their home country had managed to maintain more of these pre-migratory group memberships. In turn, this sense of social identity continuity was predictive of greater life satisfaction and fewer symptoms of depression. Nevertheless, belonging to multiple groups in Syria was also directly associated with increased depression.

### 1.3. The Present Study

Following the 2015 European migrant crisis and the implementation of the 2016 EU-Turkey agreement [34,35], Greece became a destination country for hundreds of thousands of forced migrants. According to the latest 2023 statistics, around 191,000 forced migrants are currently hosted in Greece [1], many of whom will remain in the country.

The present study aims to contribute to the existing literature by being the first to test longitudinally whether multiple group memberships before migration assist forced migrants in adjusting to Greece through both SIMIC pathways. Specifically, we designed a three-wave panel study with four-month intervals between each wave. Based on previous research, we measured three adjustment-related outcome domains [36]: sociocultural, health-related, and psychological adjustment.

**Figure 1:**
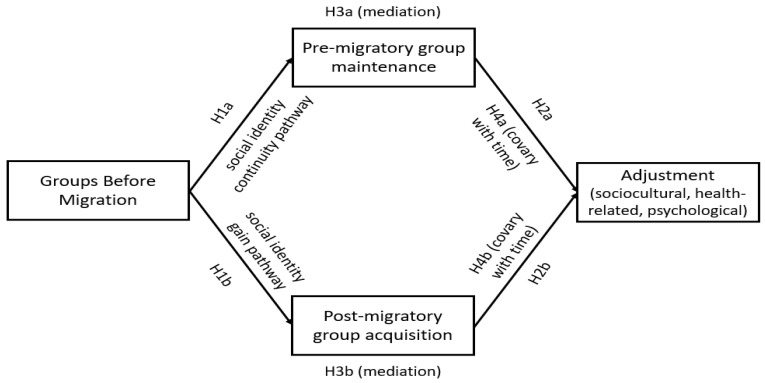
Conceptual model and hypotheses based on the two pathways of the Social Identity Model of Identity Change.

Reflecting the core predictions of SIMIC, we hypothesized that multiple group memberships before displacement would positively predict pre-migratory group maintenance (H1a) and post-migratory group acquisition (H1b) across time. We also anticipated that higher average scores of pre-migratory group maintenance (H2b) and post-migratory group acquisition (H2b) over time would contribute to better adjustment outcomes. In addition, we expected that multiple group memberships before migration would predict better average levels of adjustment over time, through group maintenance (H3a) and acquisition (H3b). Finally, we hypothesized that as group maintenance (H4a) and group acquisition (H4b) increased with time, so would adjustment. The conceptual model and the hypotheses tested are presented in Figure 1.

To a large extent, our hypotheses were confirmed. Multiple groups before displacement supported pre-migratory group maintenance and post-migratory group acquisition, which in turn predicted better adjustment-related outcomes across time. Unexpectedly, multiple groups before displacement also directly contributed to elevated levels of psychological distress symptoms. Finally, increases in group maintenance and group acquisition were associated with increases in adjustment-related outcomes over time.

## 2. Materials and Methods

### 2.1. Participants

Following Maas and Hox’s [37] guidelines regarding sample sizes in multilevel modeling (see Statistical Analyses), a minimum sample of *N* = 50 was required. A final sample of *N* = 60 was planned at baseline to allow for attrition. We recruited an opportunity sample of 60 urban sub-Saharan asylum-seekers residing in the Athens metropolitan area, who visited the social service department of an aid organization for refugees. As of mid-2023, there were approximately 20,500 refugees from sub-Saharan African countries residing in Greece [1]. This is a high-risk population, as immigrants from sub-Saharan African countries were found to have the worst adaptation outcomes among 12 ethnic/regional groups of immigrants in Greece [38].

Participants were aged between 18 to 42 years (*M*_age_ = 27.4, *SD* = 6.4), with the majority being male (*n* = 54). They were nationals of the Democratic Republic of the Congo (*n* = 21), Nigeria (*n* = 8), Cameroon (*n* = 7), Guinea (*n* = 6), Togo (*n* = 5), Sierra Leone (*n* = 4), Mali (*n* = 2), and Gambia (*n* = 2). The remaining five participants originated from South Sudan, Ivory Coast, Comoros, Benin, and Ghana. Regarding the time of arrival, 22% of the sample had been in Greece between 6 to 12 months, 55% between 1 to 2 years, and 23% over 2 years. Concerning their level of education, 25% had completed less than 9 years of formal education, 37% 9 to 12 years, and 33% more than 12 years.

### 2.2. Procedures

Data collection took place from July 2020 to July 2021. Participants arriving at the office of the aid organization were approached by the first author. They were invited to take part if they met all of the following inclusion criteria: (i) at least 18 years old, (ii) formal application for refugee protection in Greece, and (iii) ability to read and write in English and/or French. The language criterion was established because both languages were fluently spoken by the first author. Participants were excluded if they suffered from serious physical and/or mental health conditions. They all joined the study voluntarily without any incentives for participation.

Following informed consent, the researcher clarified the meaning of a social group using plain language. To verify comprehensibility, participants were asked to indicate whether four different examples (e.g., people waiting at a public service, family members) represented a social group. In the event of an incorrect response, the meaning of the term was re-explained. Subsequently, they were instructed on the meaning and usage of the visual analog response scale employed in the study (see Materials), by rating their competency or agreement according to certain casual questions (e.g., ‘Understanding and speaking Arabic’). Finally, participants completed the study questionnaires in a quiet area within the grounds of the organization. Four and eight months after their baseline measurement, each participant was invited to complete the second and third waves, respectively. The former took place in person within the same premises, while the latter was conducted online due to COVID-19 restrictions. The retention rate was 75% at T2 (*n* = 45) and 88% at T3 (*n* = 53). Almost all participants (*n* = 58) completed at least two waves.

### 2.3. Materials

For all measures, scores ranged between 1 and 9. Participants had to indicate their answers upon a 9-point horizontal visual analog scale (VAS) with anchor descriptors on the left and right. The measures for social identity and sociocultural adjustment were translated and back-translated into French from English by two bilingual translators. For the remaining questionnaires, existing translations were used.

#### 2.3.1. Social Identity Measures

Three subscales of the Exeter Identity Transition Scale [23] were adapted to the study context, to assess the three social identity variables outlined in Figure 1. For all items, the labels at the ends of the VAS were ‘disagree’ (left) and ‘agree’ (right). Multiple group membership before migration was measured by four items (e.g., ‘Before leaving my country of origin, I had strong ties with lots of different groups’). Higher scores indicated that participants felt they were part of multiple social groups prior to migration. The estimated internal consistency was α = 0.87, α = 0.84, and α = 0.93 at the three time points. Pre-migratory group maintenance was assessed using four items (e.g., ‘After coming to Greece, I still belong to the same groups I was a member of before leaving my country of origin’), with higher scores signifying that participants believed they had maintained their pre-migratory social groups after they transitioned to Greece. The estimated internal consistency of the subscale was α = 0.90, α = 0.89, and α = 0.94 at the three time points. Finally, post-migratory group acquisition was measured using four items (e.g., ‘After coming to Greece, I am friends with people from one or more new groups’), which indicated whether participants believed they belonged to new social groups after migrating to Greece. The subscale demonstrated excellent internal consistency across all three waves (α = 0.90, α = 0.93, and α = 0.97).

#### 2.3.2. Sociocultural Adjustment

The 11-item Revised Sociocultural Adaptation Scale [39] was used to assess the extent to which participants were able to display socioculturally appropriate behavior in coping with daily tasks in Greece (e.g., ‘Interacting at social events’, ‘Dealing with the bureaucracy’). The left and right labels of the VAS were ‘not at all competent’ and ‘extremely competent’. The estimates of internal consistency in each wave were α = 0.89, α = 0.91, and α = 0.91.

#### 2.3.3. Health-Related Adjustment

The 7-item physical domain subscale of the World Health Organization Quality of Life Assessment [40] (French translation [41]) was employed to assess participants’ physical health functioning and satisfaction during the last two weeks. Each item corresponded to a different facet (i.e., activities of daily living, dependence on medicinal substances and aids, energy and fatigue, mobility, pain and discomfort, sleep and rest, work capacity). The estimates of internal consistency in each wave were somewhat low but within acceptable ranges (α = 0.70, α = 0.63, and α = 0.72).

#### 2.3.4. Psychological Adjustment

We used the 10-item Hopkins Symptom Checklist-10 [42] (French translation [43]) to assess whether participants had suffered from depressive and anxiety symptomatology over the last two weeks (e.g., ‘Feeling hopeless about the future’, ‘Suddenly scared for no reason’). The anchor descriptors of the VAS were ‘not at all’ and ‘extremely’. The estimates of internal consistency in each wave were good (α = 0.85, α = 0.84, and α = 0.85). In addition, to capture the positive dimension of psychological adjustment, we assessed life satisfaction using the 5-item Satisfaction with Life Scale [44] (French translation [45]; e.g., ‘The conditions of my life are excellent’). The left and right labels of the VAS were ‘disagree’ and ‘agree’. The estimates of internal consistency in each wave were α = 0.78, α = 0.83, and α = 0.76.

### 2.4. Statistical Analyses

Given our longitudinal design, we conducted multilevel mediation analyses with time points (Level 1) nested within participants (Level 2) in order to disentangle the between- and within-person effects. In our case, between-person effects denoted the average relationship between two variables across all time points (i.e., H1–H3), whereas within-person effects reflected how this relationship changed from one time point to the next (i.e., H4).

More specifically, for each of the four adjustment outcomes, we fitted a 2-1-1 multilevel mediation model [46]. Groups before migration contained only between-person variance (i.e., as a retrospective measure, it did not make theoretical sense to change prospectively over time), while the mediators and outcomes varied with time. We decided to conduct the analyses within a multilevel modeling framework instead of a multilevel structural equation modeling framework because the former is more robust to small sample sizes [47].

To test the four models, we used the MLmed macro in IBM SPSS Statistics v. 28 with restricted maximum likelihood estimation [48,49]. The intercepts were specified as random, and the slopes as fixed. MLmed executes all the data management needed before fitting the multilevel mediation model, including the within-person centering of the Level 1 variables, and stacking the mediator and outcome variables [50]. It also provides separate estimates of the between- and within-person effects, as well as Monte Carlo 95% confidence intervals for indirect effects based on 10,000 samples [51]. The macro permits the inclusion of a maximum of three Level 2 covariates. As such, in all models, we controlled for age, gender, and time since arrival to Greece.

## 3. Results

### 3.1. Preliminary Analyses

The absolute skewness and kurtosis values of all variables at each wave lay within the acceptable ranges for normality (skewness < |2| and kurtosis < |7|; [52]). Standardized z-scores were also computed for all variables at each wave, with *z* > |3.29| being considered potential univariate outliers [53] (pp. 52–98). Only one observation regarding health-related adjustment at T1 was identified above the cut-off (*z* = 3.32). To manage this highly unlikely score, we used the winsorization substitution technique, in which the observation was replaced with the highest nonoutlier score for the health-related adjustment at T1 [54]. This was deemed a better option compared to deletion or transformation in the interest of power and interpretability, respectively. Mahalanobis distance values were also computed for all participants. A critical value signifying a potential multivariate outlier was calculated at *p* < 0.001 using a chi-square distribution with the degrees of freedom equal to the number of variables [53] (pp. 52–98); χ^2^ (21) = 46.797, *p* < 0.001. No multivariate outliers were detected according to this standard.

None of the participants had missing data on the item level. We assessed differences between those participants who completed all three waves (*n* = 40) and those who completed two (*n* = 18) or one (*n* = 2) waves by assigning a dummy variable separating these two groups. Chi-square test and *t*-test analyses (*p* < 0.05, two-tailed) revealed no significant differences between the two groups in terms of gender, age, education level, marital status, time of arrival in Greece, desire to remain in Greece, and the seven main study variables at baseline. In addition, Little’s MCAR test for all study variables was not significant, χ^2^ (1) = 0.519, *p* = 0.471, indicating that data missingness did not statistically deviate from complete randomness. Since data were likely missing completely at random, they were imputed using the expectation maximization algorithm using SPSS Missing Value Analysis [53] (pp. 374–376). This solution to missing data preserves power and it is much more robust compared to deletion, mean substitution, or regression imputation.

Finally, using Box’s *M*-test, we assessed the homogeneity of the variance-covariance matrices across the three time points. Because of the test’s sensitivity, the significance value is recommended at *p* < 0.001 [53] (pp. 210–211). Based on this threshold, Box’s *M* (97.144) was not significant (*p* = 0.002), indicating equal variance–covariance matrices across time points. The correlations among study variables can be found in Table 1.

### 3.2. Main Analyses

Table 2 presents the first (common) part of all four multilevel mediation models, with groups before migration having a significant positive fixed effect on pre-migratory group maintenance and post-migratory group acquisition (H1a, H1b). Thus, the more groups someone belonged to before fleeing their home country, the greater average levels of group maintenance and acquisition they tended to report across time. None of the control variables were significantly related to group maintenance and group acquisition.

The second part for each of the four multilevel mediation models is shown in Table 3, with groups before migration, pre-migratory group maintenance, and post-migratory group acquisition predicting each of the four outcome variables. Overall, after controlling for the effects of the two social identity pathways, groups before migration had a significant direct effect only on psychological distress. That is, multiple group memberships predicted higher average levels of distress symptoms through pathways other than social identity continuity and gain. None of the adjustment outcomes were significantly predicted by any of the control variables.

Concerning the between-person effects of the two pathways (H2a, H2b), pre-migratory group maintenance positively predicted health-related adjustment and life satisfaction, and negatively predicted psychological distress. Therefore, greater average levels of pre-migratory group maintenance predicted higher health-related adjustment and life satisfaction, and lower psychological distress. On the other hand, greater average levels of post-migratory group acquisition predicted higher sociocultural adjustment and life satisfaction.

Table 4 presents the tests of the between-person indirect effects (H3a, H3b). Groups before migration predicted higher person-average levels of health-related adjustment and lower person-average levels of psychological distress by way of person-average pre-migratory group maintenance, as well as higher person-average levels of sociocultural adjustment by way of person-average post-migratory group acquisition. For life satisfaction, the effect of groups before migration was transmitted through both group maintenance and acquisition. Therefore, multiple group memberships before fleeing supported social identity continuity and gain, which in turn resulted in better adjustment outcomes.

Concerning the within-person effects of the two pathways (H4a, H4b; Table 3), pre-migratory group maintenance positively predicted sociocultural adjustment and life satisfaction. Thus, increases in pre-migratory group maintenance predicted increases in sociocultural adjustment and life satisfaction. Similarly, increases in post-migratory group acquisition predicted increases in health-related adjustment and life satisfaction. We also reran the analyses by allowing random slopes between the mediators and the adjustment outcomes. All random effects were non-significant, indicating that within-person group maintenance and acquisition predicted the four adjustment outcomes in the same way across participants.

Finally, to compute the variance explained by our four models (Table 5), we divided the residual variance of each model by the explained variance of each respective unconditional means model [55].

## 4. Discussion

Informed by SIMIC, the current three-wave longitudinal study investigated the role of group belonging and related social identities in facilitating the transition of asylum-seekers to Greece. Specifically, it examined how multiple group belonging before migration, social identity continuity and social identity gain relate to their sociocultural adjustment, health-related adjustment, feelings of distress, and life satisfaction.

The findings were in line with the basic assumptions of SIMIC and provided evidence for the protective role of group belonging in forced migration. On the group level, multiple group memberships in their home country did not have a direct positive effect on asylum-seekers’ transition outcomes. However, the more group memberships they had before migration, the more likely they were to have preserved pre-existing social groups to assist social identity continuity and the more likely they were to have become members of new post-migratory social groups to assist social identity gain. It was through these two social identity pathways that multiple group memberships supported asylum-seekers in adjusting to Greece. Although both pathways contributed to elevated levels of life satisfaction, they also demonstrated unique effects on different aspects of asylum-seekers’ adjustment in Greece. Feelings of social identity continuity uniquely contributed to better physical functioning and health satisfaction and lower psychological distress, while social identity gain uniquely contributed to asylum-seekers’ sociocultural adjustment in Greece. According to the social identity approach to health, the primary driver of these associations is that group memberships—through their capacity to be integrated into one’s sense of self—are a source of psychological resources, including a feeling of belonging, meaning, agency, and social support [19,20,21].

Our finding on the protective role of the social identity continuity pathway for forced migrants’ life change is consistent with the results of Smeekes et al. [33] among Syrian refugees and with the results of Cruwys et al. [25] among international students. It also aligns well with evidence showing that ethnic group belonging protects forced migrants’ health and well-being by satisfying basic social identity needs, including the need for continuity [56], as well with evidence showing that ethnic community support is a determinant of happiness and better mental health among refugees [57,58]. Relatedly, the positive effect of the continuity pathway on psychological distress accords with the notion that pre-migratory group maintenance promotes post-traumatic growth following exposure to trauma and hardship [59]. Apart from the psychological resources that social identities provide, it is also possible that the maintenance of pre-migratory groups alleviates psychological distress because it reduces some of the worries and fears about the well-being and whereabouts of family members and friends [60,61].

For the first time, however, as evidenced in the context of other life transitions [24], we show that social identity gain can be equally important for forced migrants’ successful transition, especially for their life satisfaction and sociocultural adjustment. This is consistent with research demonstrating that social contact with members of the host society and a sense of host national belonging lead to increased life satisfaction and well-being [62,63], probably due to the sense of belonging to a higher status group, which results in a more positive self-concept [64]. It also aligns with research showing that the emergence of a shared refugee identity in receiving countries reduces the impact of stress on refugees’ general health by being a source of support and collective efficacy [65]. The unique contribution of the social identity gain pathway to asylum-seekers’ sociocultural adjustment appears plausible considering that developing sociocultural competence necessitates some form of interaction with new groups within larger society. Indeed, during cross-cultural transitions, it is host national identification rather than ethnic identification that facilitates sociocultural adjustment [66,67].

Contrary to our predictions, groups before migration had a direct negative impact on psychological distress. Although this deviates from previous studies on SIMIC in the context of other life transitions [22,25] and the general contention that multiple group memberships act as a social cure [19], interestingly, it replicates the finding of Smeekes et al. [33] among Syrian refugees. The authors argued that this was because being a member of multiple groups in Syria not only meant a higher probability of preserving some of these groups following resettlement (i.e., social identity continuity) but also a higher probability of being affected by the loss of valued groups (i.e., social identity discontinuity). This explanation is certainly highly possible, yet we contend that other reasons are equally likely. First, it is not merely the number of social groups that bring about the social cure, but also their characteristics. Multiple devalued and visible group memberships have been found to hinder health and well-being [68,69], a process that may be at play for forced migrants considering the stigma that many of their pre-migratory identities (e.g., ethnic, religious) carry within European societies. Second, group memberships before migration may increase psychological distress based on intragroup processes that turn the social cure into a social curse [32]. For instance, failing to meet the expectations of people back home or fears of being a burden to them may create additional stress and/or lead refugees to abstain from asking for support [70,71].

A unique contribution of the present research was its longitudinal design, which, apart from permitting more reliable cross-sectional estimates of total variable scores for each participant, allowed us to examine how changes in the two pathways related to changes in adjustment outcomes. On the within-person level, participants whose sense of continuity rose during the study experienced increases in their sociocultural adjustment and life satisfaction. Those who expanded their post-migratory group memberships over time demonstrated improvements in their health-related adjustment and life satisfaction. These results suggest that positive modifications in forced migrants’ social group memberships can be beneficial for their adjustment—even over a period of a few months. By extension, this means that brief interventions that build social identification at particular time points may offer valuable support during forced migrants’ adjustment trajectories [72].

Two findings of the within-person analyses are worth discussing. First, it seems somewhat paradoxical that increases in pre-migratory group maintenance contributed to increases in sociocultural adjustment. As already reasoned, sociocultural adjustment should mostly be affected by the social identity gain pathway, and this was the case on the between-person level. Thus, sociocultural adjustment may be realized by permanent high levels of identification with new post-migratory groups, but temporary increases in social identity continuity are also beneficial. A possible reason is that an increase in one’s sense of continuity at a specific time point facilitates sustained identification with new groups, an explanation that accords with the notion that the more strongly migrants identify with their ethnic groups, the more likely they are to engage and identify with wider society [73]. Second, psychological distress was the only outcome not related to within-person changes in social identity continuity and/or gain. As a matter of fact, the percentage of within-person variance explained in the psychological distress model was zero (see Table 5; note that there was within-person variability to explain in psychological distress over the three time points). This may be because the time course over which the effects of the two pathways on psychological distress unfold exceeds that of 8 months. In other words, had we measured participants over a longer period, we may have captured these relationships. Alternatively, the chronicity, severity, and comorbidity of mental health problems in forced migrants [3] may render their distress resistant to change—at least as a function of changes in social identification. Perhaps meaningful change in psychological distress necessitates specialized interventions for psychopathology symptoms. Obviously, the above reasonings are tentative and require further examination. Yet, what can be argued with higher certainty is that fully evaluating the effects of the two SIMIC pathways on adjusting to life change requires longitudinal designs, as their effects may not hold on both the within- and between-person levels.

In general, the present study adds to the existing evidence base on the social identity approach to health and SIMIC, by expanding its scope within a context where life change is forced and presents additional sources of stress (e.g., stigma) compared to more positive transitions. It is also one of the few instances in which SIMIC’s basic assumptions are tested with a non-WEIRD sample [30,74,75].

In addition, the study contributes to the refugee literature by testing a process-based psychological framework of forced migrants’ experiences. That is, although multiple social determinants of their adjustment have been identified ([16]; e.g., employment, family separation), this knowledge offers little insight into the psychological processes at play, thus reducing our capacity to intervene with the utmost efficiency. To illustrate this, it is not the mere fact of finding a socially respected or high-paying job that leads to optimal psychological adjustment for forced migrants [76]. More crucially, psychological adjustment is mostly related to job satisfaction, which in turn relates to the pre-migratory social identities that they bring to their new country and the post-migratory social identities that they develop [77,78]. Similarly, it is not mere contact with family members that benefits forced migrants but rather the psychological resources of family identification [79], which means that even in their physical absence, a sense of continuity and belonging may still be preserved [80].

SIMIC offers a perspective on the underlying socio-psychological processes that may protect forced migrants (i.e., social identification through the social identity continuity and gain pathways) or alternatively put forced migrants at risk, as evidenced by the direct relationship of multiple group memberships before migration to psychological distress (i.e., several processes could have contributed such as social identity discontinuity, social identity devaluation, intragroup curse processes). Therefore, our results offer specific intervention targets for policymakers, humanitarian resettlement service providers, and mental health practitioners to support forced migrants during their transition to a new land. The key point is that where forced migrants are able to cultivate meaningful group memberships and social identities through the two SIMIC pathways, they will be better off adjusting to life change. That is, efforts at the individual, community, and policy levels should be directed toward assisting forced migrants in preserving their existing, valued group memberships and social identities (e.g., by promoting close relationships with their ethnic, religious, and/or diaspora communities through funding of community centers), while simultaneously granting them the necessary resources and opportunities to join new groups and form new social identities during resettlement (e.g., by ensuring access to employment, education, and health or welfare services). In addition, interventions such as Groups 4 Health [81,82], which seek to build social identification via the two pathways, hold promise for supporting forced migrants’ adjustment by bringing about the social cure of group life. Notably, the significant within-person effects found in our study substantiate such a possibility.

### Limitations and Future Directions

The current study is not without limitations. To begin with, the small sample size may have inflated the Type I error rates or upwardly biased estimates. Nevertheless, the fact that we used restricted maximum likelihood estimation mitigates these risks to some extent [83]. Second, our findings do not necessarily generalize to the whole population of forced migrants, an issue that needs to be addressed in future research. Due to recruitment and practical barriers (e.g., lack of interpreters, lack of access to refugee camps), our sample was selected from sub-Saharan African asylum-seekers living in private housing. Relatedly, women were underrepresented in the sample because there were few arriving in the office of the aid organization. Importantly, ethnicity, visa status, place of residence, and gender are factors that may affect the observed associations in a multitude of ways. For example, for refugee women, gaining a new work-related identity in Greece may increase psychological distress because it is perceived as incompatible with the gender norms stemming from their culture, whereby they are expected to occupy domestic and caregiving roles [84]. Third, there is the issue of causality. Despite the longitudinal design and the significant within-person effects, we cannot make strong causal inferences [85]. The within-person effects of the two pathways on adjustment are not influenced by time-invariant confounders (e.g., gender, age, personality), but could be driven by the presence of unobserved time-variant confounders. For example, finding employment during the 8-month study period could have affected positively both post-migratory group acquisition and life satisfaction. Additionally, we cannot exclude the presence of cross-lagged associations from adjustment to the two pathways. For instance, it is possible that psychological distress predicts group maintenance and gain because intense feelings of distress lead participants to self-isolate. In this respect, experimental studies and longitudinal studies with baseline randomization may provide more conclusive evidence regarding the causal processes at hand. Fourth, to assess the social identity variables of SIMIC, we relied on the number of social groups to which participants felt they belonged, but we assessed neither the characteristics and interrelations among these groups nor how strongly participants identified with them. This should be a topic of further exploration since being a member of multiple social groups does not indicate automatically that these groups are distinctly represented in one’s self-concept or that one strongly identifies with them, both being necessary conditions for the social cure to emerge [19,68]. Finally, future studies may also consider potential moderators of the two social identity pathways such as social identity compatibility [22], as this would inform us on the catalysts (or boundaries) of the observed relations.

## 5. Conclusions

The current longitudinal study was the first to examine the role of the two social identity pathways of SIMIC in a population of forced migrants. Overall, our findings demonstrated that feelings of belonging to multiple social groups before migration facilitated the transition of forced migrants in Greece by functioning as a scaffold for maintaining old pre-migratory group memberships (social identity continuity pathway) and creating new post-migratory group ties (social identity gain pathway). Sustained high levels of both pathways across time and increments in both pathways over time acted as valuable resources for different aspects of their adjustment process. However, in line with the previous evidence, we found that multiple group memberships before migration can potentially act as a risk factor for their mental health. Additional longitudinal and experimental research with more representative samples of forced migrants is necessary to generalize and draw causal inferences on the observed relationships.

## Figures and Tables

**Table 1:** Correlation matrix of study variables.

Variable	1	2	3	4	5	6	7	8	9	10	11	12	13	14	15	16	17	18	19	20	21
1. GBM T1	-																				
2. GBM T2	0.53 **	-																			
3. GBM T3	0.47 **	0.35 **	-																		
4. GM T1	0.52 **	0.28 *	0.30 *	-																	
5. GM T2	0.25	0.40 *	0.06	0.51 **	-																
6. GM T3	0.28 *	0.14	0.32 *	0.64 **	0.49 **	-															
7. GA T1	0.63 **	0.29 *	0.43 **	0.58 **	0.25	0.52 **	-														
8. GA T2	0.38 **	0.59 **	0.30 *	0.31 *	0.47 **	0.36 **	0.48 **	-													
9. GA T3	0.37 **	0.15	0.49 **	0.50 **	0.33 *	0.61 **	0.53 **	0.47 **	-												
10. SA T1	0.56 **	0.47 **	0.36 **	0.62 **	0.39 **	0.37 **	0.51 **	0.41 **	0.38 **	-											
11. SA T2	0.27 *	0.59 **	0.17	0.31 *	0.52 **	0.28 *	0.27 *	0.69 **	0.25	0.59 **	-										
12. SA T3	0.24	0.27 *	0.41 **	0.35 **	0.21	0.51 **	0.44 **	0.52 **	0.46 **	0.56 **	0.54 **	-									
13. HA T1	0.18	0.18	−0.02	0.42 **	0.25	0.38 **	0.41 **	0.21	0.25	0.34 **	0.14	0.31 *	-								
14. HA T2	0.14	0.39 *	0.16	0.36 **	0.63 **	0.44 **	0.23	0.43 **	0.30 *	0.28 *	0.39 **	0.19	0.52 **	-							
15. HA T3	0.08	0.10	0.17	0.23	0.35 **	0.36 **	0.33 **	0.27 *	0.41 **	0.16	0.13	0.19	0.47 **	0.55 **	-						
16. PD T1	0.01	0.02	0.26 *	−0.19	−0.32 *	−0.36 **	−0.02	−0.13	−0.09	−0.05	−0.15	−0.15	−0.40 **	−0.29	−0.14	-					
17. PD T2	0.06	0.00	0.04	−0.16	−0.20	−0.20	−0.09	−0.32 *	−0.16	−0.14	−0.14	−0.33 *	−0.47 **	−0.41 **	−0.45 *	0.43 **	-				
18. PD T3	−0.13	0.02	0.19	−0.22	−0.34 **	−0.36 **	−0.20	−0.18	−0.17	−0.08	−0.17	−0.17	−0.37 **	−0.42 **	−0.52 **	0.57 **	0.46 **	-			
19. LS T1	0.39 **	0.19	0.07	0.51 **	0.29 *	0.30 *	0.52 **	0.44 **	0.39 **	0.45 **	0.38 **	0.27 *	0.58 **	0.38 **	0.32 *	−0.36 **	−0.36 **	−0.41 **	-		
20. LS T2	0.33 *	0.42 **	0.09	0.45 **	0.67 **	0.35 **	0.43 **	0.62 **	0.37 **	0.42 **	0.58 **	0.29 *	0.23	0.50 **	0.23	−0.23 *	−0.20	−0.31 *	0.56 **	-	
21. LS T3	0.39 **	0.22	0.27 *	0.51 **	0.39 **	0.48 **	0.49 **	0.42 **	0.60 **	0.37 **	0.34 **	0.40 **	0.38 **	0.36 **	0.40 **	−0.38 **	−0.36 **	−0.49 **	0.57 **	0.39 **	-

Note: GBM = groups before migration; GM = pre-migratory group maintenance; GA = post-migratory group acquisition; SA = sociocultural adjustment; HA = health-related adjustment; PD = psychological distress; LS = life satisfaction; * *p* < 0.05, ** *p* < 0.01.

**Table 2:** Between-person effects of groups before displacement on social identity continuity and social identity gain.

Predictor	β	SE	95% CI (LL, UL)		*p*
Fixed Effects					
Outcome: Group Maintenance					
Intercept	1.981	1.462	−0.950	4.912	0.181
Groups Before	0.445	0.131	0.182	0.708	**0.001**
Outcome: Group Acquisition					
Intercept	0.611	1.481	−2.357	3.580	0.681
Groups Before	0.733	0.133	0.467	0.999	**<0.001**

Note: Significant effects at *p* < 0.05 in bold.

**Table 3:** Fixed effects estimates for each adjustment outcome model.

	Predictor	β	SE	95% CI (LL, UL)		*p*
Sociocultural Adjustment	Fixed Effects					
	Intercept	2.997	1.060	0.871	5.123	**0.007**
	Groups Before	0.207	0.117	−0.027	0.441	0.081
	Between-Person Group Maintenance	0.210	0.115	−0.020	0.441	0.073
	Within-Person Group Maintenance	0.399	0.071	0.259	0.539	**<0.001**
	Between-Person Group Acquisition	0.246	0.113	0.018	0.473	**0.035**
	Within-Person Group Acquisition	0.108	0.056	−0.003	0.219	0.057
Health-Related Adjustment	Fixed Effects					
	Intercept	4.356	0.970	2.411	6.302	**<0.001**
	Groups Before	−0.093	0.107	−0.306	0.121	0.390
	Between-Person Group Maintenance	0.295	0.105	0.084	0.505	**0.007**
	Within-Person Group Maintenance	0.108	0.074	−0.038	0.254	0.147
	Between-Person Group Acquisition	0.190	0.104	−0.018	0.398	0.073
	Within-Person Group Acquisition	0.137	0.058	0.021	0.253	**0.021**
Psychological Distress	Fixed Effects					
	Intercept	4.440	1.161	2.112	6.769	**<0.001**
	Groups Before	0.331	0.128	0.075	0.587	**0.012**
	Between-Person Group Maintenance	−0.374	0.126	−0.627	−0.122	**0.004**
	Within-Person Group Maintenance	0.013	0.092	−0.170	0.195	0.890
	Between-Person Group Acquisition	−0.142	0.124	−0.391	0.108	0.259
	Within-Person Group Acquisition	−0.045	0.073	−0.189	0.100	0.541
Life Satisfaction	Fixed Effects					
	Intercept	2.281	0.996	0.283	4.278	**0.026**
	Groups Before	−0.083	0.110	−0.302	0.137	0.454
	Between-Person Group Maintenance	0.274	0.108	0.058	0.491	**0.014**
	Within-Person Group Maintenance	0.246	0.086	0.077	0.416	**0.005**
	Between-Person Group Acquisition	0.432	0.107	0.218	0.646	**<0.001**
	Within-Person Group Acquisition	0.164	0.068	0.030	0.299	**0.017**

Note: Significant effects at *p* < 0.05 in bold.

**Table 4:** Between-person indirect effects of groups before displacement on each adjustment outcome through group maintenance and group acquisition.

Outcome	Indirect Effects	β	95% MCCI	
			LL	UL
Sociocultural Adjustment	Group Maintenance	0.094	−0.007	0.229
	Group Acquisition	0.180	**0.013**	**0.375**
Health-Related Adjustment	Group Maintenance	0.131	**0.030**	**0.271**
	Group Acquisition	0.139	−0.012	0.312
Psychological Distress	Group Maintenance	−0.166	**−0.339**	**−0.043**
	Group Acquisition	−0.104	−0.299	0.072
Life Satisfaction	Group Maintenance	0.122	**0.022**	**0.261**
	Group Acquisition	0.317	**0.140**	**0.532**

Note: MCCI = Monte Carlo confidence interval. Indirect effects are significant when the confidence interval does not contain zero (in bold).

**Table 5:** Percentage of variance explained in each adjustment-related outcome.

Outcome	Between-Person (%)	Within-Person (%)	Total (%)
Sociocultural adjustment	45.61	28.80	38.17
Health-related adjustment	36.01	7.59	21.48
Psychological distress	35.93	0.00	16.60
Life satisfaction	63.76	14.17	39.59

## Data Availability

The data presented in this study are available on request from the corresponding author. The data are not publicly available due to restrictions of privacy.
